# Clinical outcomes of three treatment methods for humeral shaft fractures: a comparative study

**DOI:** 10.3389/fsurg.2026.1738549

**Published:** 2026-03-13

**Authors:** Feng Wang, Feng Xie, Ximing Liu, Guodong Wang, Wei Wang

**Affiliations:** 1 Department of Orthopedic Surgery, Hubei Provincial Hospital of Traditional Chinese Medicine, Hubei Shizhen Laboratory, Affiliated Hospital of Hubei University of Chinese Medicine, Wuhan, China; 2 Department of Orthopedics Surgery, Jiangxia District Hospital of Traditional Chinese Medicine, Wuhan, China; 3 Department of Orthopedic Surgery, General Hospital of Central Theater Command, Wuhan, China

**Keywords:** curative effects, external fixator, humerus shaft fracture, nonoperative treatment, open reduction and plate osteosynthesis

## Abstract

**Objective:**

To compare the clinical efficacy of three techniques (nonoperation, external fixation, and open reduction and plate osteosynthesis) for the treatment of diaphyseal fractures of the humerus, thereby providing guidance for the selection of treatment methods.

**Methods:**

A retrospective analysis was conducted on 138 patients with humeral shaft fractures who received treatment at the Department of Orthopedics and Traumatology, Hubei Provincial Hospital of Traditional Chinese Medicine between January 2021 and December 2024, all with complete follow-up data. The patients were divided into three groups based on their treatment methods: the nonoperative treatment (NOT) group, the external fixation (EF) group, and the open reduction and plate osteosynthesis (ORPO) group. In the NOT group, 46 patients received small splint fixation/plaster/functional bracing. There were 28 patients in the EF group and 64 patients in the ORPO group. The patients were followed up regularly at outpatient appointments or by telephone. The follow-up evaluations included measurements of fracture-healing time, fracture-healing rate, postoperative complications, length of hospital stay, and time to return to work after treatment. Furthermore, the clinical outcomes included the Constant shoulder score, the Mayo elbow score, and patient satisfaction at the last follow-up.

**Results:**

The mean follow-up durations for the NOT, EF, and ORPO groups were 21.4 ± 3.7, 20.1 ± 4.7, and 22.6 ± 5.4 months, respectively. The mean union times for the NOT, EF, and ORPO groups were 9.0 (8.0–11.25) months, 10.0 (9.0–12.0) months, and 12.0 (10.25–12.0) months, respectively; additionally, the mean union rates were 95.7%, 96.4%, and 96.9%, respectively. There were significant differences in union time among the three groups (*P* = 0.002, *ε*^2^ = 0.079), but the magnitude of the difference was limited. The main complications were significantly different among the three groups (*P* < 0.001, V = .438). Residual deformity/malunion was the main complication in the NOT group, while postoperative radial nerve palsy and infection were the main complications in the EF and ORPO groups, respectively. The length of hospital stay for the three groups was 4.0 (2.0–5.25) days, 10.5 (5.0–12.0) days, and 11.0 (8.0–15.0) days, respectively. The time to return to work after treatment for the three groups was 13.0 (10.0–16.0) weeks, 16.0 (14.25–18.75) weeks, and 14.0 (10.0–17.0) weeks, respectively. The mean duration of hospital stay and number of days away from work were significantly lower in the NOT group than in the other two groups (*P* *<* *0.05*), Although the hospitalization time in the EF group was shorter than that in the ORPO group, the time from treatment to return to work was longer, with both differences being statistically significant (*P* < 0.05). At the last follow-up, the postoperative Constant scores and Mayo scores were not significantly different among the three groups (*P* *>* *0.05*). Patient satisfaction also differed significantly among groups, with higher satisfaction in the NOT group than in the EF and ORPO groups (*P* < 0.05, *η*^2^ = .046), although the effect size was small.

**Conclusions:**

Within the limitations of this retrospective, non-randomized study, our findings suggest that all three treatment modalities achieve comparable fracture union rates and functional outcomes in humeral shaft fractures. Meanwhile, these results underscore the value of individualized treatment selection. In appropriately selected patients, NOT remains a viable and cost-effective option. But these findings should be validated in prospective trials.

## 1 Introduction

Humeral shaft fracture is a common type of fracture in clinical practice that occurs from 2 cm below the surgical neck of the humerus to 2 cm supracondylar. Humeral shaft fractures account for 1%–5% of all body fractures and approximately 20% (1, 2) of humeral fractures. The incidence of fractures is highest among young men (21∼30 years old) and elderly women (60∼80 years old) (3). Owing to rapid industrialization in China and the gradual ageing of society, the incidence of humeral shaft fractures has increased, thereby placing a considerable economic burden on societies and families.

There are many treatment options for humeral shaft fractures among adults. Nonoperative treatment (NOT) includes casts, splints, and functional braces. NOT for humeral shaft fractures has been reported to result in a fracture healing rate (4) of more than 90%. However, some studies have shown that NOT still has a 2% to 10% fracture nonunion rate and a high rate of malunion (5). Recently, the use of ORPO, including dynamic compression plating (DCP), locking compression plating (LCP), minimally invasive plate osteosynthesis (MIPO), and intramedullary nail internal fixation, to treat humeral shaft fractures has gradually increased. Although most of the literature reports satisfactory therapeutic effects, this technique requires a larger incision. Extensive soft tissue dissection, iatrogenic nerve injury, secondary extraction, and internal fixation are inevitable (6). External fixation (EF) has been used to treat humeral shaft fractures in recent years. Although it is not the mainstream treatment at present, several studies have shown that EF can achieve the same therapeutic effect as open reduction and internal fixation in the treatment of humeral shaft fractures. EF is also considered an effective and feasible treatment for humeral shaft fractures (7, 8). Although there are many evidence-based domestic and international studies comparing the efficacy of various treatments as well as meta-analyses comparing different treatment approaches, there is still considerable controversy regarding the optimal treatment for humeral shaft fractures among adults. Previous studies have been largely confined to pairwise comparisons, lacking direct and comprehensive comparisons among all three approaches, especially in terms of patient-reported outcomes such as satisfaction and economic indicators. This study aimed to compare the clinical outcomes of these three treatments for humeral shaft fractures, thereby providing guidance for the selection of clinical treatments.

## 2 Materials and methods

### 2.1 Inclusion and exclusion criteria

#### 2.1.1 Inclusion and exclusion criteria

The inclusion criteria were as follows: patients aged ≥18 years old; free movement of the shoulder and elbow before injury; humeral shaft fracture was limited to one side, without shoulder or elbow injury; the time interval between fracture and treatment was < 2 weeks; and the follow-up time was ≥12 months.

The exclusion criteria were as follows: bilateral humeral shaft fracture; previous history of shoulder and elbow dysfunction; combined shoulder or elbow injury or fracture; combined with ipsilateral forearm fracture; open fracture, or nerve or vascular injury; multiple injuries with systemic damage to multiple organs; pathological fracture; old fracture; severe medical conditions that preclude surgery; mental illness precluding cooperation with treatment; or poor compliance.

### 2.2 General information

In this study, 138 patients (72 males and 66 females) with humeral shaft fractures, with average age of 39.1 years (18–72 years), received treatment at the Bone Injury Diagnosis and Treatment Center of Hubei Hospital of Traditional Chinese Medicine from January 2021 to December 2024. Follow-up data were also collected. According to the AO/OTA classification, there were 68 type A fractures (A1-15, A2-32, A3-21), 47 type B fractures (B1-19, B2-15, B3-13), and 23 type C fractures (C1-16, C2-7). The time from injury to treatment ranged from 2 h to 12 days, with a mean of 5.8 days. The patients were divided into three groups. The NOT group achieved functional healing through closed reduction and external fixation (46 patients, including 23 patients with splint fixation, 7 patients with plaster fixation and 16 patients with functional support fixation), EF group (28 patients), and ORPO group which achieved anatomical reduction and rigid internal fixation through open surgery (64 patients, including 19 DCP patients, 34 LCP patients, and 11 MIPO patients). All treatments were performed by the same team of physicians.

This study was approved by the Theoretical Committee. Although this was a retrospective observational study, we also adhered to the Declaration of Helsinki and relevant policies in China. We reached a consensus with all participants, and all patients signed informed consent forms.

### 2.3 Preoperative preparation

Full-length humerus x-rays were routinely taken for all patients after admission, and plain CT scans and 3D reconstructions were performed for some patients with complex fractures. All patients were temporarily immobilized with plaster or splints, during which the combined injury was treated and the underlying diseases were managed. Some nonsurgical patients underwent manual reduction and external fixation of fractures in the outpatient department without special preoperative preparation.

### 2.4 Treatment methods

#### 2.4.1 Nonsurgical treatment

After closed manual reduction, a small splint/plaster/functional support was used for fixation. Within 4 weeks after fixation, the external fixation was reviewed once a week and dynamically adjusted based on reductions in limb swelling and fracture displacement; thereafter, it was reviewed once every 2 weeks. Functional exercise was emphasized in each review during this period. The reduction criteria were as follows (8): anterior-posterior angle < 20°, internal and external turning angles < 30°, rotation deformity < 15°, and shortening < 3 cm.

#### 2.4.2 Treatment with an external fixator

Perform routine surgical preparation. According to the fracture form and displacement characteristics, the fracture was rectified under the guidance of the C-arm, and the needle entry point was determined after anatomic reduction. The needle entry points were generally 2–4 cm and 8 cm from the fracture end. An external fixator was installed (Comfort Company, China), and C-arm fluoroscopy was performed again to confirm that there was no significant displacement. If any residual displacement was found after the installation of the external fixer, it was fine-tuned through the external fixer. When reduction was difficult, a small incision was made locally at the fracture site, reduction was carried out under direct vision, the needle was pierced, and the support was installed.

#### 2.4.3 Treatment of internal fixation with a steel plate

An operation was scheduled after systemic stability. DCP/LCP (Comfort Company, China)/MIPO intraplate fixation was performed according to the principles of internal fixation for fracture treatment.

### 2.5 Postoperative management

Postoperative follow-up and evaluation criteria: All patients required regular outpatient review before fracture healing. The surgical treatment group was reviewed at 2, 4, 8, 12, and 16 weeks after treatment; thereafter, follow-up assessments were performed every 2∼3 months. Fracture healing patients were reviewed every 6 months. Parameters such as operation time, fracture healing time, complications, indirect economic cost (length of stay (hospital stay for outpatient patients was recorded as 0 days), and time to return to work after treatment were collected and compared. At the last follow-up, the Constant score and Mayo score were used to evaluate shoulder and elbow joint function in patients. A self-administered questionnaire was used to evaluate patients’ overall satisfaction with the treatment process and outcome. The satisfaction assessment employed a 100-point scale, which was referenced against a 5-point Likert scale: 1–20 = “Very dissatisfied,” 21–40 = “Dissatisfied,” 41–60 = “Neutral,” 61–80 = “Satisfied,” and 81–100 = “Very satisfied.” All statistical analyses and functional evaluations were performed independently by 2 doctors who were not involved in treatment or operations in this study.

The criterion for fracture healing was no tenderness or percussion pain at the fracture end. X-rays revealed callus formation or encapsulation of at least 3 cortical bones in the anterior–lateral position, absence of fracture space or callus passage. Delayed union was defined as fracture union after more than 6 months, and nonunion was defined as fracture failure if no sign of callus growth was found on imaging examination after 2 months of continuous observation (9). The malunion criteria were as follows (8): anterior-posterior angle >20°, and/or internal and external turning angle > 30°, and/or rotation deformity > 15°, and/or shortening > 3 cm.

### 2.6 Statistical analysis

All statistical analyses were performed using SPSS software (version 26.0; IBM Corp., Armonk, NY, USA). Continuous variables were assessed for normality using the Shapiro–Wilk test. Normally distributed data are presented as mean ± standard deviation (SD), whereas non-normally distributed data are summarized as median with interquartile range (IQR). Categorical variables are expressed as frequencies and percentages.

Between-group comparisons for continuous variables were conducted using one-way analysis of variance (ANOVA) or the Kruskal–Wallis test, as appropriate. Categorical variables were compared using the chi-square test or Fisher's exact test when indicated. Exact *P*-values are reported, and a two-sided *P* value < 0.05 was considered statistically significant.

To quantify the magnitude of between-group differences, effect sizes were calculated and reported alongside *P* values. For categorical outcomes, Cramer's V was used as a measure of effect size, while eta-squared (*η*^2^) or partial eta-squared was reported for continuous outcomes where applicable. Where applicable, effect estimates are presented with 95% confidence intervals (CIs).

## 3 Results

The baseline characteristics of patients in the three treatment groups are presented in Table 1. No statistically significant differences were observed among the groups with respect to age, sex, cause of injury, AO/OTC fracture classification, or duration of follow-up.

**Table 1:** Baseline characteristics of patients in the three treatment groups.

Variable	NOT group (*n* = 46)	EF group (*n* = 28)	ORPO group (*n* = 64)	*P* value
Sex, *n* (%)				0.79
Male	24 (52.2)	13 (46.4)	35 (54.7)	
Female	22 (47.8)	15 (53.6)	29 (45.3)	
Age, years	38.9 ± 15.2	36.8 ± 12.0	40.4 ± 14.2	0.63
Cause of injury, *n* (%)				0.97
Traffic accident	20 (43.5)	12 (42.9)	30 (46.9)	
Fall	22 (47.8)	14 (50.0)	28 (43.8)	
Heavy object impact	4 (8.7)	2 (7.1)	6 (9.4)	
AO/OTC fracture classification, *n* (%)				0.88
Type A	27 (58.7)	14 (50.0)	35 (54.7)	
Type B	15 (32.6)	9 (32.1)	21 (32.8)	
Type C	4 (8.7)	5 (17.9)	9 (14.1)	
Duration of follow-up, months	21.4 ± 3.7	20.1 ± 4.7	22.6 ± 5.4	0.08

Values are presented as mean ± SD or *n* (%).

*P* values were calculated using one-way ANOVA or the chi-square test, as appropriate.

A statistically significant difference in fracture healing time was observed among the three groups (*P* = 0.002). However, the associated effect size was moderate (*ε*^2^ = 0.079), suggesting that the magnitude of the difference was limited. Regarding postoperative complications, the incidence of malunion differed significantly among the three groups, with the highest rate observed in the NOT group and the lowest in the ORPO group (*P* < 0.001), The magnitude of the association between treatment modality and malunion was large, as indicated by a Cramer's V of 0.438, suggesting a clinically meaningful difference in malunion rates among the three treatment strategies. Other complications, including infection, delayed or non-healing, and new radial nerve injury, were descriptively reported. The main complication in the NOT group was malunion (55.6%), with an angulation deformity < 20°. The main complications in the EF group were postoperative infection and malunion, whereas those in the ORPO group were postoperative infection and radial nerve injury, as shown in Table 2. The postoperative infection in the EF and ORPO groups was healed by dressing changes or debridement and symptomatic treatment with antibiotics. There was no loosening or rupture of the external fixator or internal fixator in either group. In addition to 2 patients with radial nerve palsy who failed to recover during the observation period after surgery, 1 patient in the EF group was confirmed by second-stage exploration to have a partial tear and degeneration, and 1 patient in the ORPO group was broken, all of whom received reparative treatment. The remaining patients recovered spontaneously within 2 to 5 months. Bone nonunion in all three groups healed after open reduction, combined with autogenous iliac bone graft and LCP internal fixation. The external fixator was removed after fracture healing in both the NOT group and the EF group. During follow-up, internal fixation plates were removed in 28 patients in the ORPO group. No refractures occurred in the three groups during follow-up.

**Table 2:** Comparison of postoperative conditions among the three groups.

Variable	NOT group (*n* = 46)	EF group (*n* = 28)	ORPO group (*n* = 64)	*p*	Effect size
Fracture healing time (weeks)^†^	9.0 (8.0–11.25)^b^	10.0 (9.0–12.0)^b^	12.0 (10.25–12.0)^a^	.002	*ε*^2^ = .079
Fracture healing rate (%)	95.7	96.4	96.9		
Postoperative Complications					
Infection [cases (%)]	1 (2.2)	4 (14.3)	7 (10.9)		
Delayed healing [ex. (%)]	3 (6.5)	3 (10.7)	2 (3.1)		
Non-healing [ex. (%)]	2 (4.3)	1 (3.6)	2 (3.1)		
Postoperative new radial nerve stimulation or injury [cases (%)]	2 (4.3)	2 (7.1)	7 (10.9)		
Malunion, *n* (%)	25 (54.3)^a^	9 (32.1)^b^	6 (9.4)^c^	<.001	V = .438
Economic cost					
Length of hospital stay (days)^†^	4.0 (2.0–5.25)^b^	10.5 (5.0–12.0)^a^	11.0 (8.0–15.0)^a^	<.001	*ε*^2^ = .556
Time to return to work (weeks)^†^	13.0 (10.0–16.0)^b^	16.0 (14.25–18.75)^a^	14.0 (10.0–17.0)^b^	.010	*ε*^2^ = .054
Joint function score					
Constant score^‡^	88.9 ± 3.7	87.6 ± 5.3	87.3 ± 5.1	.237	*η*^2^ = .021
Mayo score^‡^	94.6 ± 3.7	92.9 ± 4.5	92.9 ± 5.2	.124	*η*^2^ = .030
Patient satisfaction (%)^‡^	88.1 ± 8.1^a^	84.8 ± 10.2^b^	85.3 ± 9.8^b^	.031	*η*^2^ = .046

Different superscript letters indicate statistically significant differences between groups (*p* < .05).

Effect sizes are reported as epsilon squared (*ε*^2^), eta squared (*η*^2^), or Cramer's V (V).

† Data are presented as median (interquartile range) and analyzed using the Kruskal–Wallis test.

‡ Data are presented as mean ± standard deviation and analyzed using one-way analysis of variance.

Length of hospital stay also differed significantly among groups (*P* < 0.001), with a large effect size (*ε*^2^ = 0.556), indicating substantial between-group variability. In addition, time to return to work showed a statistically significant difference (*P* = 0.010); the time to return to work after treatment in the EF group was significantly longer than in the ORPO and NOT groups, although the corresponding effect size was small to moderate (*ε*^2^ = 0.054). See Table 2.

**Figure 1:**
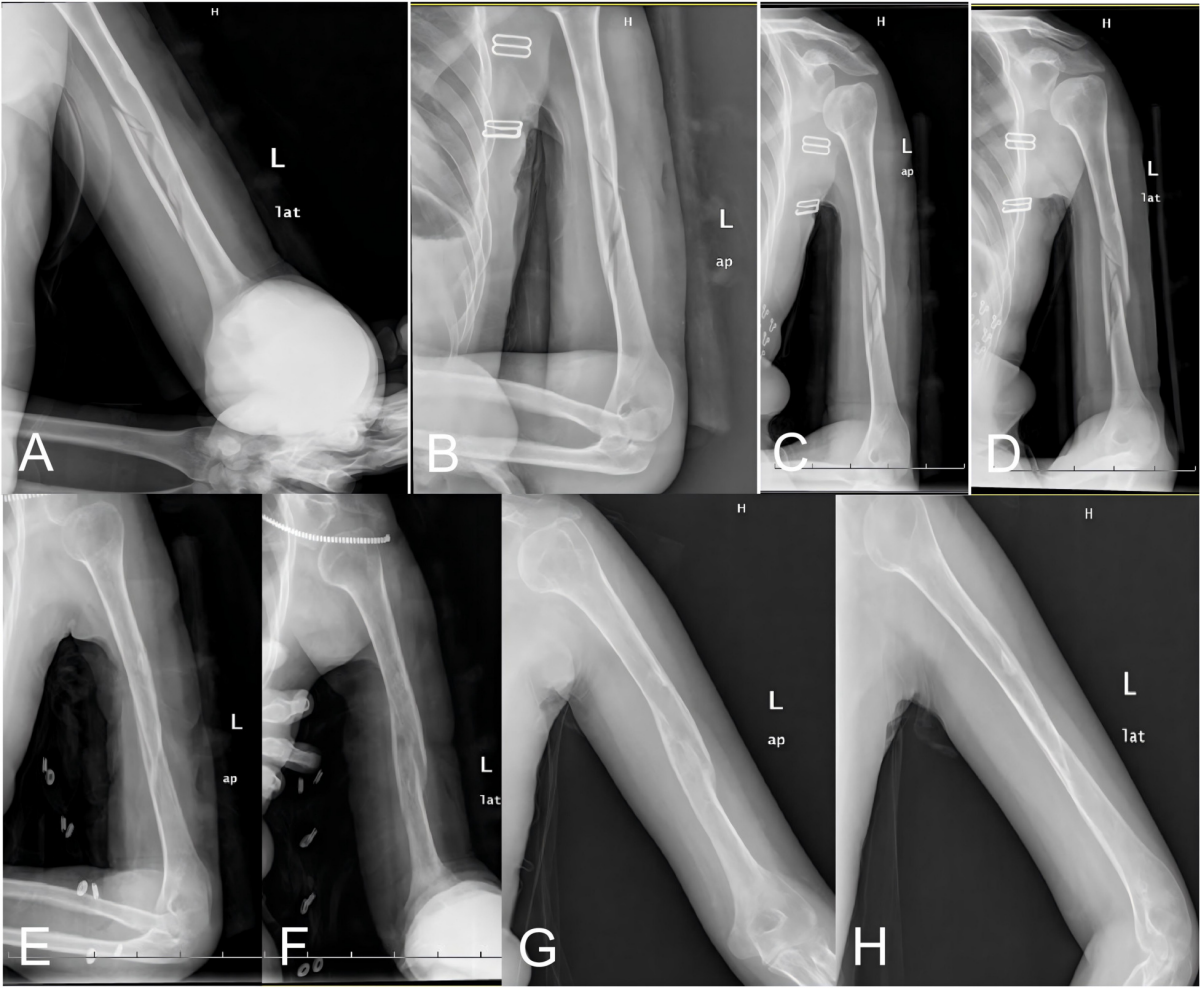
35-year-old female with a left humeral shaft fracture (AO/OTA C1). **(A,B)** Initial anteroposterior and lateral radiographs. **(C,D)** Post-reduction radiographs following splint application. **(E,F)** Follow-up radiographs at 10 weeks demonstrating callus formation. **(G,H)** Final follow-up radiographs showing cortical continuity and medullary canal patency.

**Figure 2:**
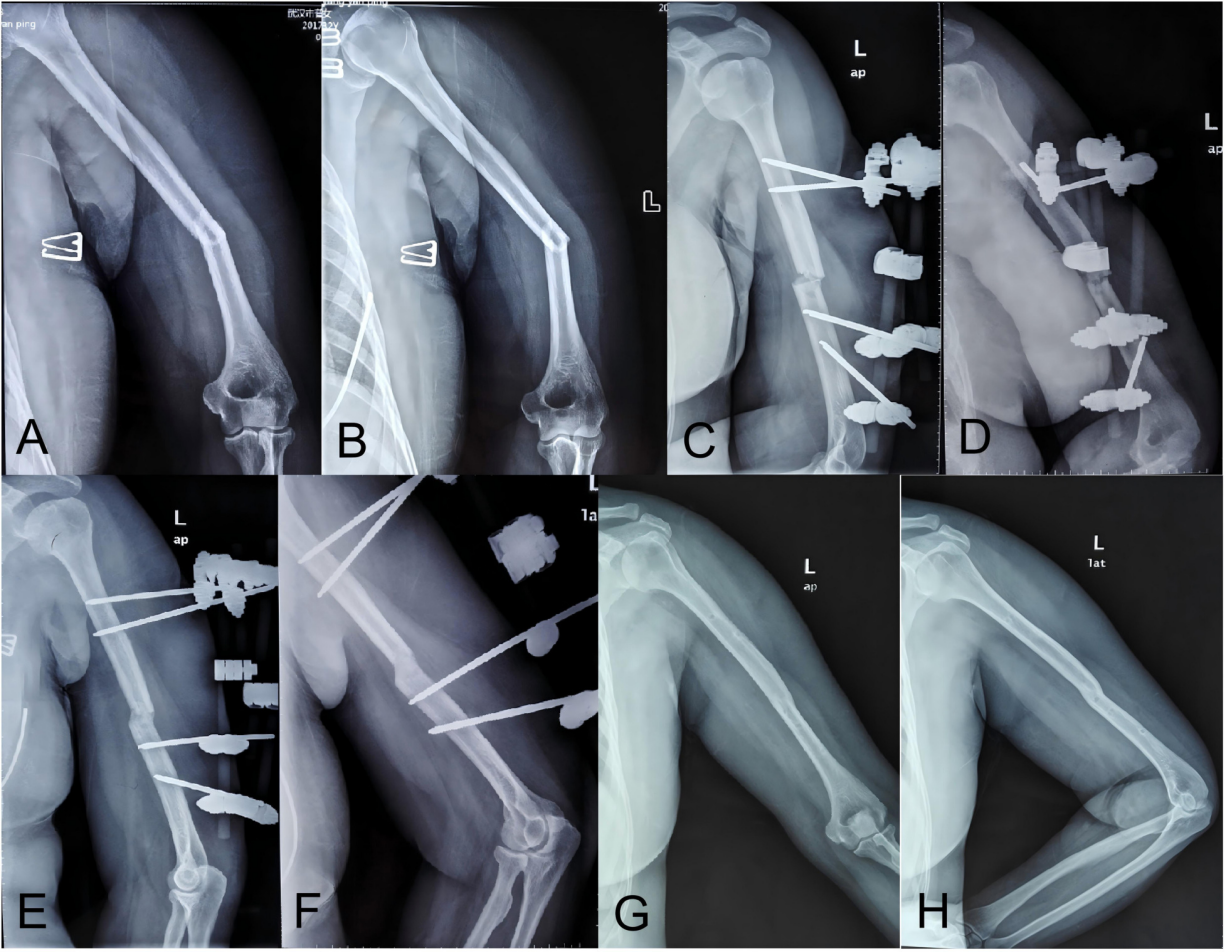
32-year-old female with closed left humeral shaft fracture (AO/OTA A3). (A,B) Initial anteroposterior and lateral radiographs. (C,D) Post-closed reduction EF. (E,F) 11-week follow-up showing union; fixator removed. (G,H) Final follow-up radiographs showing cortical continuity and medullary canal patency.

**Figure 3:**
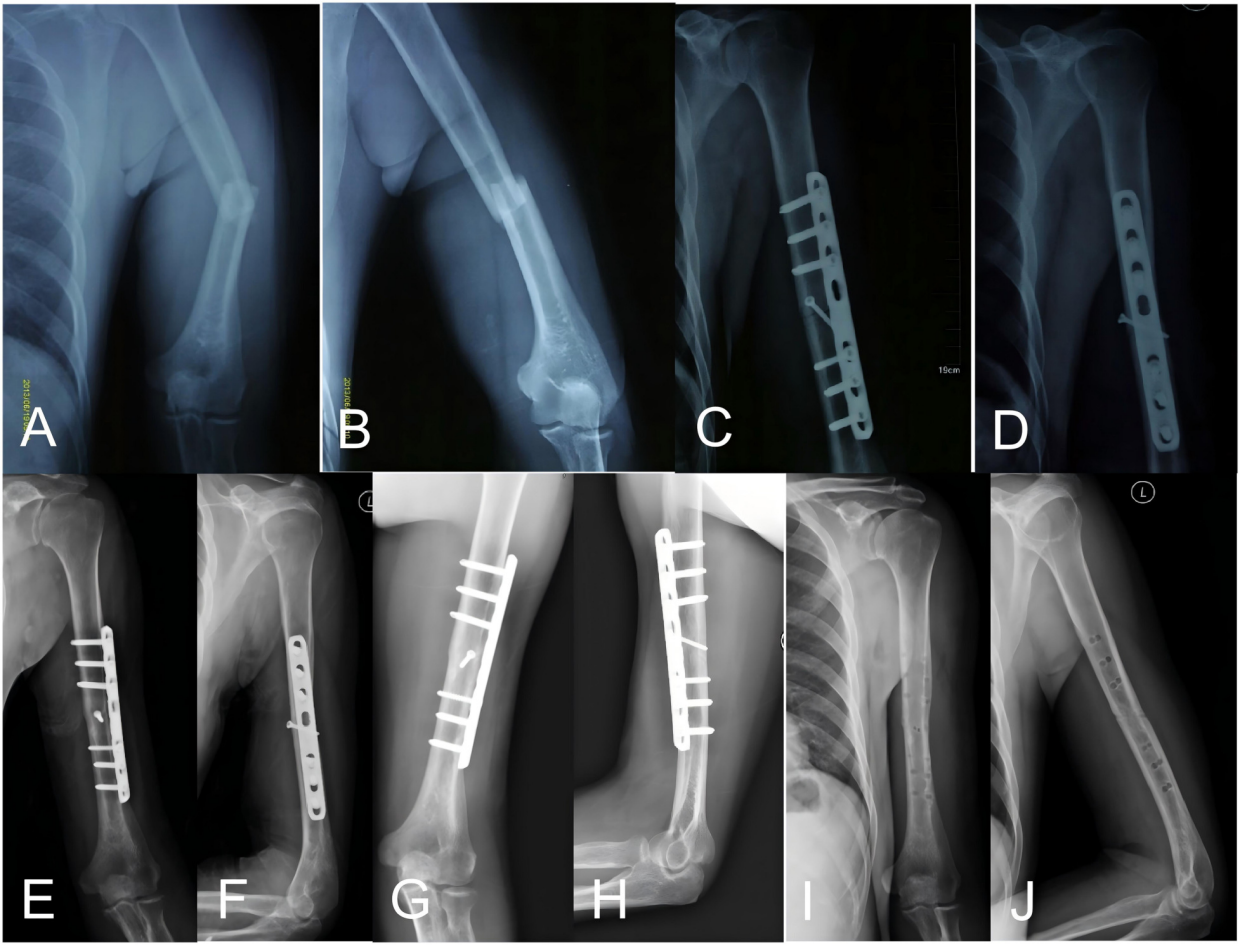
49-year-old male with a left humeral shaft fracture (AO/OTA A3) with angular shortening. **(A,B)** Preoperative radiographs. **(C,D)** Immediate postoperative radiographs after ORPO. **(E,F)** Radiographs at 12 weeks following surgery. **(G,H)** Radiographs at 24 months post-surgery. **(I,J)** Radiographs after removal of the internal fixation.

No significant differences were found among the three groups in Constant score (*P* = 0.237, *η*^2^ = 0.021) or Mayo score (*P* = 0.124, *η*^2^ = 0.030), and effect size estimates indicated minimal between-group differences in functional outcomes. At the last follow-up, patient satisfaction also differed significantly among groups, with higher satisfaction in the NOT group than in the EF and ORPO groups (*P* < 0.05, *η*^2^ = .046). This indicates that, despite similar levels of functional recovery across the three groups after treatment, the NOT group demonstrated lower indirect economic costs and higher overall satisfaction. Typical cases are shown in Figures 1–3.

## 4 Discussion

There is an ongoing debate over the optimal treatment modality for humeral shaft fractures. As early as 1964, Bohler argued against surgical treatment of humeral shaft fractures, suggesting that “conservative treatment” was preferable (10). Sargeant et al. (5) suggested that, in most cases, humeral shaft fractures can be treated nonsurgically, and for any nonunion that results, plate fixation should be used, which can achieve a high rate of bone healing. Papasoulis et al. (11) verified the efficacy of nonsurgical treatment for humeral shaft fractures by performing a meta-analysis of 18 clinical studies; they reported that the mean healing time of conservative treatment for humeral shaft fractures was 10.7 weeks, the healing rate was 94.5%, and the functional recovery of the affected limb was satisfactory. Domestic scholars also reported that the treatment of humeral shaft fractures with closed reduction, small splints, and plaster external fixation achieved satisfactory results (12, 13). Owing to the advantages of simple operation, ease of adaptation, and early mobilization of the shoulder and elbow joints, nonsurgical treatment remains widely recognized and used.

External fixators are used for the treatment of humeral shaft fractures. In the early stage, these fixators are used only as a temporary fixation method for patients with multiple injuries, open humeral fractures, poor soft-tissue conditions, or damage control in emergencies. However, with the widespread application of external fixator technology in the treatment of long tubular bone fractures in recent years, its unique therapeutic advantages have gradually been recognized (14, 15). Scaglione et al. (8) reported that 85 patients with humeral shaft fractures were treated with external fixators, and the results revealed that the fracture healing rate was 97.6%, the average healing time was 12 weeks, the delayed healing rate was 1.2%, the malunion rate was 7.2%, and 3 patients experienced postoperative nail hole infection. The patients recovered well after the operation. The authors concluded that an external fixator is an effective treatment for humeral shaft fractures, providing fracture-end reduction and stable fixation, and is the ultimate fixation method for these fractures.

The use of ORPO for the treatment of humeral shaft fractures enables direct anatomic reduction, compression fixation of the fracture end, ionization and exploration of the radial nerve, and early postoperative functional rehabilitation (16). This treatment approach was once regarded as the gold standard for humeral shaft fracture treatment (17). Therefore, some studies have suggested that ORPO is better than nonsurgical treatment for managing humeral shaft fractures, and it remains the most popular treatment. The results of the current study seem to reflect this phenomenon, as the number of patients in the ORPO group was greater than that in the other treatment groups. DCP, LCP, and MIPO are common internal fixation techniques for treating humeral shaft fractures. Although their technical advantages vary slightly, all can achieve good clinical outcomes. We classify them into the ORPO group for macroscopic comparison.

Therefore, all three treatments can be used for the treatment of humeral shaft fractures, but there is no consensus regarding the optimal treatment. To further clarify the clinical efficacy of nonsurgical vs. surgical treatment for humeral shaft fractures, Mahabier et al. (18) reviewed the data of 186 patients with humeral shaft fractures (91 vs. 95 patients who did not undergo surgery). The results revealed that the median healing time was 11 to 28 weeks, suggesting that the healing time and complication rate of the two treatments were similar. A recent prospective randomized controlled study by Matsunaga et al. (19) also supported this view. A comparison of the clinical efficacy of plate internal fixation with nonsurgical treatment for humeral shaft fractures revealed that the incidence of bone disunion (0% vs. 15%) and the degree of deformity union (internal and external angulation) in the plate internal fixation group were significantly greater than those in the treatment group (2° vs. 10.5°) as well as the nonsurgical treatment group. There were no statistically significant differences in the DASH, SF-36, pain and Constant scores, etc. The findings of the current study are consistent with these previous results, as there were no statistically significant differences in the mean fracture healing time, healing rate, complication rate, Constant score of the shoulder joint, or Mayo score of the elbow among the NOT, EF, and ORPO groups. These studies suggest that nonsurgical treatment is as effective as surgical treatment for managing humeral shaft fractures.

Notably, the major complications of humeral shaft fractures vary depending on the treatment modality. In this study, nonsurgical treatment was mainly functional reduction, resulting in a higher incidence of bone disunion and malunion, whereas the incidence of surgery-related complications was greater in the EF and ORPO groups. These results are similar to those of Papasoulis et al. (11). Another study reported that residual (>10°) sagittal angulation is possible in more than 10% of patients with humeral shaft fractures after conservative treatment and that residual coronal angulation (>10°) can reach 20% (11, 20). Kapil Mani et al. (21) reported that 108 patients with humeral shaft fractures were treated conservatively. The mean healing time was 12.2 weeks, the healing rate was 97.2%, the incidence of radial nerve injury was 5.5%, and the incidence of malunion (<15°) was 90.9%, but this did not affect the joint function of the patients satisfactorily. Therefore, the humerus is likely a non-weight-bearing bone of the upper limb surrounded by abundant soft tissue wrap, and the upper and lower joints have a relatively large range of motion. Thus, the humerus can accept angulation deformities within a certain range without affecting the function and beauty of adjacent joints. Shields et al. (20) suggested that residual deformities within a certain range (sagittal angle ≤18°, coronal angle ≤27°) after conservative treatment of humeral shaft fractures do not affect patients’ subjective functional scores. Sargeant et al. (5) found no correlation between fracture malunion and functional scores. However, this idea needs further testing. In contrast, for the surgical treatment of humeral shaft fractures, the literature reports that the nonunion rate ranges from 0% to 13%, the radial nerve injury rate ranges from 3% to 29%, and the deep infection rate is approximately 3.5% (22). The incidence of complications is even higher following the removal of secondary internal fixation (23).

The three methods (small splint, cast, brace) used in this study are commonly used in the clinical nonsurgical treatment of humeral shaft fractures. All are non-invasive treatment modalities that adhere to the principles of functional therapy. Many studies have reported that one of these methods alone can also achieve satisfactory results in the treatment of humeral shaft fractures. There is no strict difference in the treatment of humeral shaft fractures between these three fixation methods; therefore, in this study, they were grouped together as “nonsurgical treatment”. In our experience, each of these three nonsurgical EF methods has advantages and disadvantages in clinical application. For example, owing to axillary obstruction, minor splint fixation is not suitable for 1/3 or more fractures of the upper humeral shaft, and it needs to be adjusted frequently during treatment. Cast fixation requires hyperarticulation, restricts the movement of the adjacent shoulder and elbow joints during fixation, and is less permeable during fixation. Additionally, this fixation method is less stable for oblique fractures of the humeral shaft. Similar to the principle of splint fixation, functional support can be carried out early via shoulder and elbow joint functional exercise, as well as soft tissue pressure on the upper arm to indirectly maintain fracture stability. Furthermore, air permeability is better than that of plaster fixation. Multiple reports in the literature have reported good results from these methods of functional support, but the prices are relatively high. Additionally, for overly obese people, these support methods are not suitable for use because excessive soft tissue wrapping of the upper arm will cover the deformity of the humerus. Notably, the choice of nonsurgical treatment in this study is also influenced by the patient's preferences, tolerance, level of cooperation, economic conditions, and aesthetics. For example, more complex and unstable fractures are more likely to be included in the surgical group (EF or ORPO), while patients in favorable clinical conditions but with poor economic status are more likely to be assigned to the NOT group. Furthermore, treatment selection is also influenced by the doctor's preferences and habits.

Although this study revealed that the function of the affected limb after the three treatments was similar, the final postoperative satisfaction was highest in the NOT group. The reasons for this finding may be related to lower treatment costs in this group of patients. The expected better clinical outcomes in nonsurgical patients, which are aesthetically acceptable even if the anatomic site is not fully healed, may be due to lower treatment costs, shorter hospital stays, and shorter time to return to work after treatment, resulting in greater patient satisfaction with the outcome. However, such considerations necessitate a formal cost-effectiveness analysis for verification. Based on this study, the authors conclude that NOT is an important and viable management option for closed humeral shaft fractures. For failure of conservative treatment, an open fracture, or a complex fracture type, surgery (external fixator/internal fixation, etc.) is recommended based on the situation. Notably, for nonsurgical treatment of humeral shaft fractures, patient cooperation is crucial, and patients who are unable to cooperate with or comply with treatment are also at increased risk of nonunion or complications.

This retrospective study has inherent limitations. First, the non-randomized design introduces selection bias, as treatment choice was influenced by fracture complexity, surgeon judgment, and patient factors, potentially affecting outcome comparisons. Second, we could not perform multivariable adjustments for important unmeasured confounders such as smoking status, exact fracture displacement, and occupational demands, which may influence healing and return-to-work time. Third, economic comparisons based on hospital stays and self-reported return-to-work are preliminary and lack a formal cost-effectiveness analysis. Fourth, heterogeneity within groups (e.g., different splinting/plating techniques) may mask technique-specific effects. Finally, the intermediate-term follow-up and absence of patient-reported outcome measures limit the assessment of long-term quality of life. Despite these limitations, our study provides a real-world comparison of three treatment strategies and demonstrates that all three treatment modalities achieved comparable fracture union rates and functional outcomes for humeral shaft fractures. In secondary outcomes, NOT was associated with significantly shorter hospital stays and earlier return to work, but also a higher incidence of malunion, while surgical approaches (EF and ORPO) carried procedure-specific risks, including postoperative infection and radial nerve injury. These results suggest that treatment selection should be individualized, taking into account factors such as fracture pattern, patient preferences, functional demands, and tolerance for procedural risks. NOT remains a viable and cost-effective option for selected patients, particularly those with closed, stable fractures and good compliance. Future prospective, randomized controlled trials are warranted to validate these findings and further elucidate the long-term outcomes and cost-effectiveness of each approach.

## Data Availability

The original contributions presented in the study are included in the article/Supplementary Material, further inquiries can be directed to the corresponding author.

## Supplementary material

Supplementary File 1 Relevant clinical data supporting the findings of this study was provided.
